# Two Rare Cases of Neonatal Meningitis Caused by Globicatella sanguinis at the Marrakech University Hospital: Diagnosis and Therapeutic Implications

**DOI:** 10.7759/cureus.110754

**Published:** 2026-06-12

**Authors:** Achraf Janni, Fahem Mohamed Aziz, Taoufik Ben Houmich, Asmae Lamrani Hanchi, Nabila Soraa

**Affiliations:** 1 Department of Microbiology, Faculty of Medicine and Pharmacy of Marrakesh, Cadi Ayyad University, Mohammed VI University Hospital of Marrakesh, Marrakesh, MAR

**Keywords:** antibiotic resistance, beta-lactams, globicatella sanguinis, maldi-tof, meningitis

## Abstract

Two infants, aged 3 and 5 months, were admitted to the neurosurgery ward with febrile meningeal signs and underwent lumbar punctures, which revealed turbid cerebrospinal fluid (CSF) with a predominantly neutrophilic pleocytosis, decreased CSF glucose levels, and elevated CSF protein levels. CSF cultures revealed alpha-hemolytic colonies. Identification by time-of-flight mass spectrometry with matrix-assisted laser desorption and ionization (MALDI-TOF) confirmed *Globicatella sanguinis (G.* *sanguinis)* in both cases.* G. sanguinis *is a pathogenic bacterium that is rarely isolated in clinical practice and can be misidentified as alpha-hemolytic streptococci by conventional laboratory methods. Central nervous system infections caused by *G. sanguinis* in infants are uncommon but potentially life-threatening. Blood cultures remained negative.

Antimicrobial susceptibility testing was performed by microdilution to determine MICs according to the European Committee on Antimicrobial Susceptibility Testing and the Susceptibility Testing Committee of the French Society of Microbiology (CASFM-EUCAST) guidelines for viridans group streptococci/*Streptococcus pneumoniae*. In the absence of standardized guidelines specific to *G. sanguinis*, interpretation was based on these criteria. The isolate showed decreased susceptibility to beta-lactams and retained susceptibility to glycopeptides. Both infants received beta-lactam antibiotics. One had a favorable clinical outcome, whereas the other had a fatal outcome. These findings highlight the value of advanced identification methods (MALDI-TOF) in preventing misidentification and optimizing antibiotic therapy.

## Introduction

Bacterial meningitis in children remains a medical emergency, with significant morbidity and mortality, particularly in settings where access to rapid diagnosis and intensive care is limited. Early and appropriate treatment is crucial for improving prognosis [1,2].

*G. sanguinis *is a rare opportunistic pathogen composed of Gram-positive, facultatively anaerobic, catalase-negative cocci that has been sporadically reported in three cases of bacteremia in newborns and in one case of central nervous system infection in infants, according to a literature review of 21 articles published between 2001 and 2025 [3-5]. Phenotypically, it may resemble an alpha-hemolytic streptococcus, which may result in misidentification when using conventional diagnostic methods [6,7]. *G. sanguinis* can present atypical antibiotic susceptibility, including reduced susceptibility to some beta-lactams, a first-line antibiotic for pediatric meningitis. We report two cases of *G. sanguinis* meningitis diagnosed at the Marrakech University Hospital, highlighting diagnostic challenges and therapeutic implications.

## Case presentation

Case 1

A three-month-old male infant with a history of spina bifida, repaired on day 12 of life, and major congenital triventricular hydrocephalus secondary to stenosis of the aqueduct of Sylvius, underwent endoscopic ventriculocisternostomy at two months of age. Three days after the procedure, he developed fever, vomiting, and poor feeding.

On physical examination, the infant presented with a fever of 39°C, accompanied by drowsiness, axial and peripheral hypotonia, and a bulging fontanelle. The head circumference was 48 cm (>97th percentile) for a weight of 7.3 kg, within normal limits for age. The infant was hemodynamically stable, with no signs of acute dehydration. The pupils were equal and reactive.

Laboratory investigations (Table 1) revealed a marked systemic inflammatory response, characterized by an elevated C-reactive protein (CRP) level of 186 mg/L and leukocytosis (16.76 × 10⁹/L) with neutrophilia (9.82 × 10⁹/L). A hypochromic microcytic anemia was also noted, with a hemoglobin level of 7.2 g/dL, a mean corpuscular hemoglobin (MCH) of 25.4 pg, and a mean corpuscular volume (MCV) of 77.8 fL. Lumbar puncture revealed turbid CSF with pleocytosis (478 leukocytes/mm³) and neutrophil predominance (90%). Protein concentration was elevated (2 g/L), and CSF glucose was markedly decreased (0.05 g/L), with a concurrent capillary blood glucose level of 0.9 g/L. 

**Table 1:** Laboratory test results with corresponding reference ranges. CRP: C-reactive protein, WBC: White blood cells, MCV: Mean corpuscular volume, MCH: Mean corpuscular hemoglobin, CSF: Cerebrospinal fluid

Parameter	Result	Reference Range
CRP (C-Reactive Protein)	186 mg/L	< 5 mg/L
Leukocytes (Total WBC)	16.76 × 10⁹/L	6.0 – 18.0 × 10⁹/L
Neutrophils	9.82 × 10⁹/L	1 – 6.0 × 10⁹/L
Hemoglobin	7.2 g/dL	9.5 – 14.1 g/dL
MCV (Mean Corpuscular Volume)	77.8 fL	68 – 108 fL
MCH (Mean Corpuscular Hemoglobin)	25.4 pg	24 – 35 pg
CSF Cells	478/mm³	< 5/mm³
CSF Protein	2 g/L	0.10 – 0.45 g/L
CSF Glucose	0.05 g/L	2/3 of blood glucose levels (0.59 g/L)

**Figure 1:**
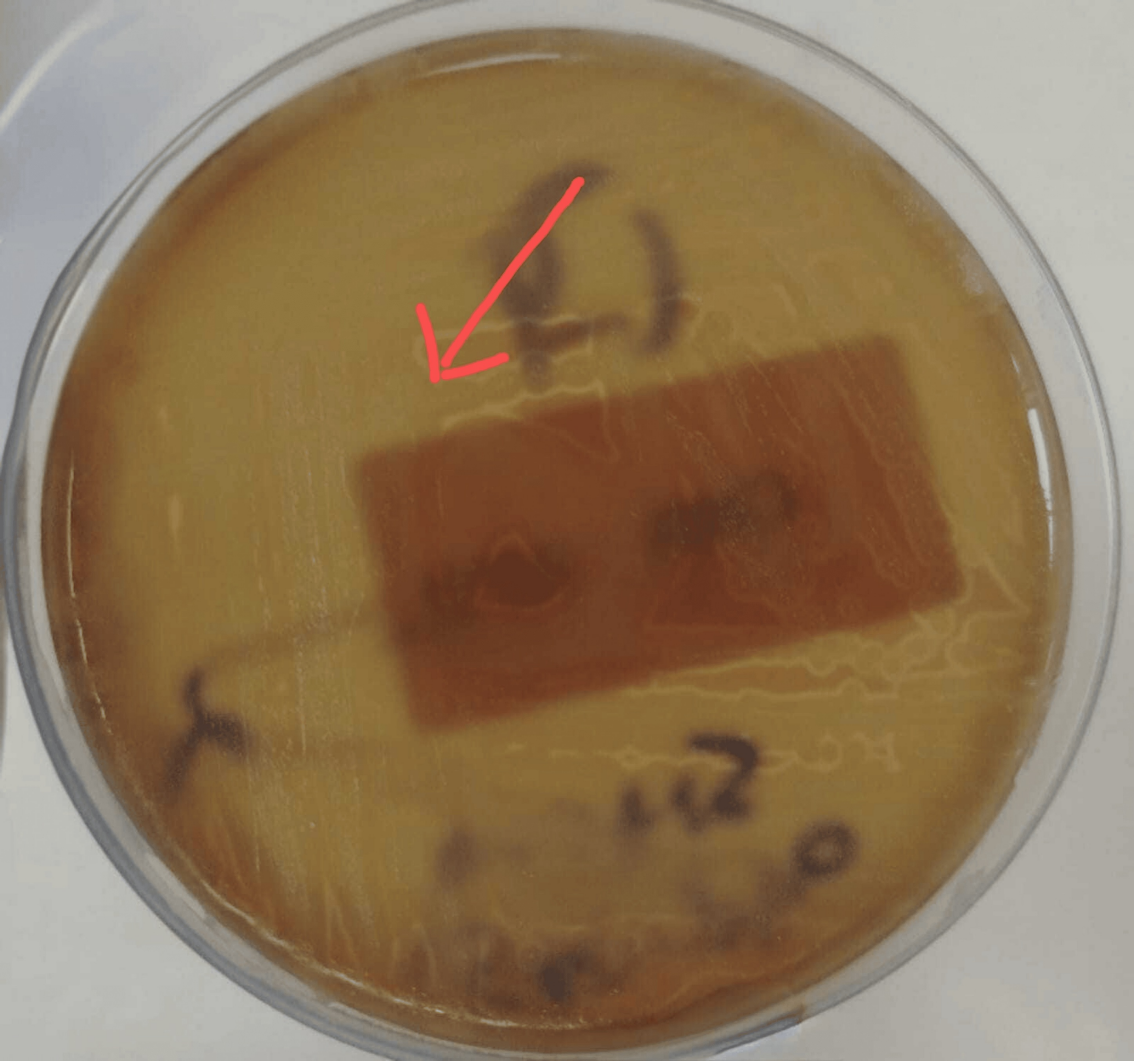
Colony morphology of G. sanguinis on blood agar.

After 48 hours of incubation at 37°C, alpha-hemolytic colonies were observed (Figure 1). Identification by *MALDI-TOF* confirmed the organism as *G. sanguinis, *with a score of 99.99%, using database version V3.3*.* The BioFire FilmArray® multiplex PCR assay did not detect any pathogens included in the meningitis/encephalitis panel. Blood cultures (BD BACTEC™ system) remained negative after five days of incubation.

Antimicrobial susceptibility testing was performed using the Vitek-2 system with the ST03 card, in accordance with the manufacturer’s instructions and the European Committee on Antimicrobial Susceptibility Testing and the Susceptibility Testing Committee of the French Society of Microbiology (CASFM-EUCAST) guidelines for viridans group streptococci/*Streptococcus pneumoniae *[8]. Due to the absence of standardized guidelines specific to *G. sanguinis*, and according to the literature, interpretation was based on these criteria [5,8]. The antibiogram (Table 2) revealed decreased susceptibility to beta-lactams (penicillin G: MIC > 0.25 mg/L; ceftriaxone: MIC = 2 mg/L; cefotaxime: MIC = 0.6 mg/L), fluoroquinolones (levofloxacin: MIC > 16 mg/L; moxifloxacin: MIC = 2 mg/L), and erythromycin (MIC = 0.5 mg/L), while retaining susceptibility to vancomycin (MIC = 0.12 mg/L), teicoplanin (MIC = 1 mg/L), and linezolid (MIC = 2 mg/L).

**Table 2:** Antibiogram results and corresponding MICs.

Antibiotic	Result	MIC (mg/L)
Penicillin G	Resistant	> 0.25 (>0,06)
Cefotaxime	Resistant	0.6(> 0,5)
Ceftriaxone	Resistant	2 (>0,5)
Levofloxacin	Resistant	> 16 (>2)
Moxifloxacin	Resistant	2 (>0,5)
Vancomycin	Susceptible	≤0.12 (≤ 2)
Teicoplanin	Susceptible	≤0.5 (≤ 2)
Linezolid	Susceptible	≤2 (≤ 2)
Erythromycin	Resistant	0.5 (> 0,25)

Empirical antibiotic therapy with ceftriaxone (100 mg/kg/day) was initiated intravenously. Partial clinical improvement was observed on day four, despite persistent fever; CRP remained elevated at 70 mg/L. The addition of vancomycin led to defervescence and a gradual normalization of inflammatory markers (CRP decreased to 8 mg/L).

Case 2

Three years later, a five-month-old female infant, being followed for a posterior fossa tumor, was admitted to the neurosurgery ward (both cases were treated in the same ward) with fever, vomiting, seizures, and poor feeding. Vaccinations were up to date according to the national immunization schedule [9].

On admission, the physical examination revealed a temperature of 39°C, axial and peripheral hypotonia, lethargy, tachycardia (168 beats per minute), tachypnea (61 breaths per minute), and hypotension (77/38 mmHg). Signs of dehydration (sunken eyes, dry tongue) were noted. The pupils were equal and reactive to light [9].

Laboratory results (Table 3) showed an elevated CRP (96.8 mg/L), leukocytosis of 29.54 × 10⁹/L with neutrophilia (19.67 × 10⁹/L), and anemia (hemoglobin 8.5 g/dL). Lumbar puncture revealed turbid CSF with marked pleocytosis (2,400 cells/mm³), predominantly neutrophils (70%). Biochemical analysis of the CSF revealed elevated protein (0.67 g/L) and markedly decreased glucose (0.007 g/L), with a concurrent capillary blood glucose of 0.73 g/L [9]. 

**Table 3:** Laboratory test results and the associated reference ranges. CRP: C-reactive protein, CSF: Cerebrospinal fluid

Parameter	Result	Reference Range
CRP	96.8 mg/L	< 5 mg/L
Leukocytes	29.54× 10⁹/L	6.0 – 18.0 × 10⁹/L
Neutrophils	19.67 × 10⁹/L	1 – 8.5 × 10⁹/L
Hemoglobin	8.5 g/dL	9.5 – 14.1 g/dL
CSF Cells	2400/mm³	< 5/mm³
CSF Protein	0.67 g/L	0.10 – 0.45 g/L
CSF Glucose	0.007g/L	2/3 of blood glucose levels ( 0.48 g/L)

The culture yielded alpha-hemolytic colonies, and identification by *MALDI-TOF *confirmed *G. sanguinis, *with a score of 99.99%, using database version V3.3. The BioFire FilmArray® multiplex PCR did not detect any of the pathogens included in the meningitis/encephalitis panel. Blood cultures (BD BACTEC™ system) remained negative after five days of incubation [9].

An automated susceptibility testing panel was performed to determine MICs by microdilution according to CASFM-EUCAST guidelines for viridans group streptococci/*Streptococcus pneumoniae* [10]. In the absence of specific guidelines for *G. sanguinis*, due to the absence of standardized guidelines specific to G. sanguinis, and according to the literature, results were interpreted using these criteria [5]. The antibiogram (Table 4) showed reduced susceptibility to penicillin G, ampicillin, cefotaxime, and ceftriaxone (ceftriaxone MIC = 0.75 mg/L), and susceptibility to levofloxacin, moxifloxacin, vancomycin, teicoplanin, linezolid, erythromycin, clindamycin, and gentamicin [9].

**Table 4:** Antibiogram results of the isolated bacterial strain.

Antibiotic	Result	MIC (mg/L)
Penicillin G	Resistant	0.5 (>0,064)
Ampicillin	Resistant	> 0.5 (>0,5)
Cefotaxime	Resistant	1.5 (>0,5)
Ceftriaxone	Resistant	0.75 (>0,5)
Levofloxacin	Susceptible	≤0.001 (≤ 0,001)
Moxifloxacin	Susceptible	0.25 (≤ 0,5)
Vancomycin	Susceptible	0.15 (≤ 2)
Teicoplanin	Susceptible	0.25 (≤ 2)
Linezolid	Susceptible	1 (≤ 2)
Erythromycin	Susceptible	0.20 (≤ 0,25)
Clindamycin	Susceptible	0.10 (≤ 0,5)
Gentamicin	Susceptible	115 (≤ 256)

Empirical combination antibiotic therapy with ceftriaxone (100 mg/kg/day) and gentamicin (3 mg/kg/day) was initiated, along with rehydration and close neurological monitoring. The patient’s clinical condition deteriorated rapidly before susceptibility results were available, necessitating transfer to the pediatric intensive care unit. Despite intensive supportive care, she developed hemorrhagic shock complicated by cardiopulmonary arrest leading to death within two days [8].

## Discussion

*G. Sanguinis* is a rare bacterium belonging to the phylum Firmicutes and the family Aerococcaceae. In 1992, Collins et al. described a new genus, *Globicatella*, and a new species, *G. sanguinis*, by differentiating human strains of *Streptococcus uberis* based on 16S rRNA sequencing and phenotypic characteristics [11,12].

To date, this genus comprises two species, *G. sanguinis* and *G. sulfidifaciens*, which are gram-positive, facultatively anaerobic, alpha-hemolytic, and catalase-negative cocci [13]. Although *G. sanguinis* is considered a member of the human commensal flora, it has been recognized as an opportunistic pathogen associated with bacteremia, urinary tract infections, meningitis, and endocarditis [14,15]. Urine, wound/device-related, and repeat CSF cultures were not systematically performed. Therefore, the source of infection could not be established, representing a limitation of this case series.

From an epidemiological perspective, reported human infections remain sporadic. They have been reported predominantly in high-risk individuals including those at the extremes of age, those with immunocompromising conditions, or those undergoing invasive procedures or surgery [4,9,14-18]. However, causality cannot be established from this small case series.

In the present cases, both infants were hospitalized for treatment in the neurosurgery department, underscoring the importance of strict aseptic techniques during surgical procedures and close clinical monitoring of high-risk patients [5]. Given the long interval of three years between the admission of the two infants and the absence of temporal clustering, the probability of nosocomial transmission is low.

A major challenge is the risk of misidentification. In culture, *G. sanguinis* can be confused with viridans group streptococci due to similar phenotypic characteristics, including alpha-hemolytic colonies, catalase negativity, and optochin resistance. Phenotypic identification methods are often limited or unavailable, which contributes to underdetection. In this context, *MALDI-TOF* is a key diagnostic tool, and in cases of diagnostic uncertainty, 16S rRNA gene sequencing may be considered [5,19]. However, we considered the absence of 16S rRNA sequencing confirmation to be a relatively minor limitation, given that the MALDI-TOF identification score reached 99.99%.

This has important clinical implications, as *G. sanguinis* exhibits an atypical susceptibility profile, with occasionally elevated MICs for certain beta-lactam antibiotics, particularly ceftriaxone, which is often used as first-line therapy for pediatric meningitis [20]. These data highlight the importance of accurate identification of the isolate, timely communication of results to the clinician, and early adjustment of antibiotic therapy guided by MICs. The lack of standardized guidelines specific to *G. sanguinis* remains a limitation, requiring cautious interpretation and, ideally, multidisciplinary discussion between clinicians and microbiologists. In both infants, the isolates showed reduced susceptibility to beta-lactams while remaining susceptible to glycopeptides (vancomycin, teicoplanin) and linezolid.

Finally, the small number of published cases and the lack of standardization in antimicrobial susceptibility testing methods limit direct comparisons. The reporting of new cases helps improve recognition of this pathogen and better define its antimicrobial susceptibility profiles and risk factors.

## Conclusions

These two observations suggest the pathogenic potential of *G. sanguinis* to cause severe meningitis in infants, particularly in high-risk situations and in the context of neurosurgical procedures. However, the small sample size, the lack of molecular confirmation by 16S rRNA sequencing, and the limited epidemiological investigation are insufficient to establish a specific tendency for severe meningitis following neurosurgical procedures. Identification by *MALDI-TOF* helps reduce the risk of misidentification. In the absence of antimicrobial susceptibility guidelines specific to *G. sanguinis* and given the reported reduced susceptibility to beta-lactams, antimicrobial therapy should be adjusted early according to microbiological findings and MICs. Strengthening reporting systems and systematically collecting clinical (treatment timeline, clinical outcome...) and microbiological (MALDI-TOF score, molecular confirmation, MIC method...) data are crucial to establishing diagnostic and therapeutic guidelines.
